# Hepatic Duct Division During Robotic Living Donor Hepatectomy: A Comparison Between the Novel Triple C (Clip–Clamp–Cut) and the Cut–Suture Techniques

**DOI:** 10.1155/2024/8955970

**Published:** 2024-10-15

**Authors:** Arvinder S. Soin, Kamal S. Yadav, Fysal Valappil, Nikhitha Shetty, Raghav Bansal, Suchet Chaudhary, Ankur Gupta, Amit Rastogi, Prashant Bhangui

**Affiliations:** Institute of Liver Transplantation and Regenerative Medicine, Medanta The Medicity Hospital, Gurugram, India

## Abstract

**Background:** In robotic donor hepatectomy (RDH), hepatic duct division (HDD) and its stump closure technique are of paramount importance in avoiding postoperative biliary complications in both donors and recipients. We describe our novel triple C (“clip–clamp–cut”) technique of HDD.

**Methods:** Out of 4016 living donor liver transplant (LDLT) (2004–October 2023), we have performed 208 RDH cases since December 2019. This study is a retrospective analysis of the first 160 RDH cases. After excluding the first 20 RDH cases (learning curve) and 3 left-sided RDH cases, 137 cases with no exclusion criteria were included. We divided these 137 donors into the “cut and suture” (CS) group (*n* = 33) and the “triple C” technique group (*n* = 104). We compared intraoperative details and postoperative outcomes.

**Results:** All 137 robotic donors and 128/137 recipients are currently well. Donor biliary leak rate was significantly lower among the triple C group (*n* = 3, 2.9%) compared to the CS group (*n* = 5, 15.2%) (*p*=0.009). No other differences in postdonation morbidity were observed among the two groups. Recipient biliary complication rate was lower in the triple C group than in the CS group although not statistically significant (10.6% vs. 15.1%; *p*=0.537), despite more multiple biliary anastomoses in the former. No significant differences in post-transplant recipient morbidity and mortality were observed.

**Conclusions:** Our simple yet novel triple C technique enables clean, precise, bloodless HDD resulting in lower donor and potentially recipient biliary complication rates. The ease and reproducibility make it ideal for widespread adoption.

## 1. Introduction

Robotic donor hepatectomy (RDH) is being increasingly adopted at living donor liver transplant (LDLT) centers given its advantages with better cosmesis and reduced scar-related problems for the liver donor [1–3]. However, some technical surgical issues continue to be safety concerns, especially for the new adopters negotiating their surgical learning curve. The graft HD division is one such step. With the currently described “cut and suture” (CS) or “clip and cut” [4] techniques, there is usually brisk arterial bleeding from the duct edge(s) and the hilar plate which requires control with pressure, diathermy, or suturing. The bleeding obscures the surgical view, and it or the measures to control it may compromise the donor or recipient HD stump length, the duct edge(s), or lumen, especially in complex biliary anatomy in right lobe (RL) grafts. This may, in part, be responsible for the higher incidence of biliary complications often reported in the initial series of minimally invasive surgery LDLT [5].

We report a simple “clip–clamp–cut” (triple C) technique that ensures a bloodless field during graft HD transection, thus allowing the surgeon a clear view and safe duct transection.

The accompanying video demonstrates the triple C technique, while this manuscript carries its detailed description, and a comparison of the donor and recipient outcomes between this technique and the CS method employed in the earlier part of our series.

## 2. Methods

Out of a total of 4016 LDLTs, we have performed 208 RDH cases since December. This study is a retrospective analysis of prospectively maintained data of the first 160 consecutive RDH out of a total of 208 performed since December 2019 with a minimum of 5 months of follow-up. All these LDLTs were ABO-compatible. During the learning phase of RDH (initial 20 RDH cases), we excluded donors with complex graft (≥ 2 right portal veins [RPVs], ≥ 2 right hepatic arteries [RHAs], ≥ 2 right HDs [RHDs], significant right inferior hepatic vein [RIHV] [> 5 mm], graft weight > 800 g, graft-to-recipient weight ratio [GRWR] < 0.8, and remnant < 33%) or with history of previous upper abdominal surgery (cholecystectomy). The first 20 cases that were considered to be a part of the early learning curve and three left-sided RDH were excluded from the analysis. After the initial 20 RDH cases, we offered RDH to all-comer donors as we became familiar with the robotic platform and it simulates open technique the closest.

**Figure 1:**
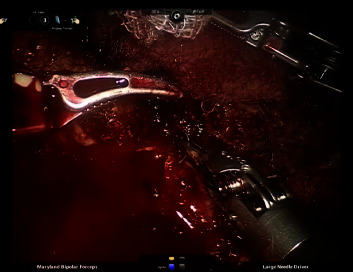
Cut–suture (C-S) technique: hemorrhagic field during HD division.

Among the remaining 137 total robotic right donor hepatectomy (RRDH) cases, the RHD transection was done in the first 33 cases by the CS group technique (Figure 1) and in the remaining 104 cases, by the triple C technique. The Clavien–Dindo classification was used for grading complications in donors and recipients [6]. The same surgical team performed all the operations in both groups. The study group was followed up for a mean of 14 months (5–34 months).

The study was approved by the Institutional Review Board-MICR 1651/2023.

### 2.1. Donor and Recipient Evaluation Protocol

#### 2.1.1. Donor Selection and Evaluation

All donors were spouses, or first- or second-degree relatives aged 18–55 years, with body mass index (BMI) ≤ 30. They underwent a detailed systemic and psychiatric evaluation, a triple-phase dynamic contrast-enhanced CT scan for fat estimation, vascular anatomy and volumetry, selective liver biopsy, and 6-step counseling including a session with an independent donor advocate.

#### 2.1.2. Recipient Evaluation

All recipients underwent a standard liver, systemic, and psychiatric evaluation.

### 2.2. Surgical Technique

Our operative protocol of total RRDH using the triple C technique of hepatic duct division (HDD) is described below.

Using a five-port (four robotic and one laparoscopic) technique, the RL is mobilized off the diaphragm and inferior vena cava (IVC), and the right hepatic vein (RHV) is isolated, following which a silicon Foley catheter is passed medial to it. Then, cholecystectomy is done. The RPV and RHA are dissected and temporarily clamped to mark the transection line. Liver transection is done with a harmonic scalpel, bipolar forceps, suture ligation, and clips.

**Figure 2:**
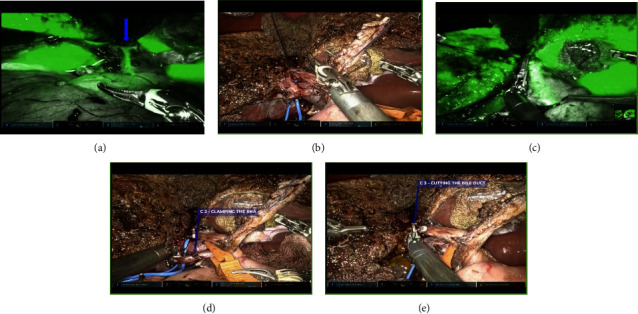
Novel “triple C (clip–clamp–cut) technique: (a) ICG delineating biliary confluence (arrow), (b) looping of right hepatic duct, (c) clipping of right hepatic duct, (d) clamping of right hepatic artery, and (e) cutting right hepatic duct.

After 70% liver transection, the triple C technique (Figure 2) is employed as demonstrated in the accompanying video clip (Supporting Information) and described below.

*The triple C technique*: Prior to the HD transection, the RHA is dissected off the posterior and lateral surfaces of the RHD. The hepatic duct confluence (HDC) and the right border of the RHD are identified. After 70% transection, the caudate lobe is transected, and a PDS thread (P1) is passed from the apex of the transected caudate up and anteriorly to emerge superior to the hilar plate.

The remaining liver tissue is cleared off the HDC with bipolar forceps. ICG camera is used to confirm the HDC and the RHD takeoff multiple times. After retracting the HDC anteriorly, a Maryland forceps is used to make a rent in the hilar plate slightly posteriorly and beyond the apex of the HDC and is passed posteroanteriorly to isolate the RHD which is then slung with another PDS thread (P2) which emerges between the HDC and the hilar plate. The RHD is held up with P2 and clipped with a Hem-o-lok® clip (*clip*-C) leaving a safe stump on the donor side. A “safety” robotic clip (size 100) is applied to the right of the Hem-o-lok® clip to secure it and prevent its slippage. The RHA is temporarily clamped (*clamp*-C). The RHD is cut (*cut*-C) to the right of the robotic clip. The previously placed P1 is used to sling up the remaining hilar plate which is then clipped with another Hem-o-lok® clip, a safety robotic clip on its right on the donor side and a laparoscopic clip (200/300) on the graft side, and divided. In the case of two RHDs over 4 mm apart (Huang Type 3 or 4 biliary anatomy), two separate Hem-o-lok® clips may be needed. The lower end of the previously placed silicon catheter is taken from below upwards, emerging superior to the PV bifurcation. The hanging maneuver is thus established for the remaining transection. The RHA clamp is now released after a clamp time of 5-6 min.

The rest of the transection is completed with the hanging maneuver. A 9- to 10-cm Pfannenstiel incision is made, and a 15-cm endo-bag is inserted. After placing the RL in the endo-bag, the RHA is Hem-o-lok® clipped and divided. The RPV is divided between Hem-o-lok® clip plus two safety robotic clips on the left and a laparoscopic (400) clip on the right side. The RHV is divided with 45-mm endo GIA stapler, and the graft is retrieved.

Modifications to the current triple C technique are occasionally needed. For instance, when there are two RHDs that are 4 mm or closer, a single Hem-o-lok® clip may not be safe to apply. In that case, the RHA is clamped, the first RHD is CS, and then the second RHD is clipped and then cut as described above.

## 3. Statistical Methods

The analysis included profiling of patients on different demographic, clinical, and laboratory parameters. Descriptive analysis of quantitative parameters was expressed as means and standard deviation. Categorical data were expressed as absolute number and percentage. Independent Student *t*-test was used for testing of mean between two independent groups. Cross tables were generated, and the chi-square test was used for testing of associations. *P* value < 0.05 was considered statistically significant. All analyses were done using SPSS software, version 24.0.

## 4. Results

### 4.1. Study Population

In the 137 consecutive RRDH cases in the study cohort, there were 33 and 104 cases in the CS and triple C groups, respectively. The demographic parameters (age, sex, and BMI) of the donors and recipients in both groups were well matched (Tables 1a and 1b).

### 4.2. Donor Perioperative Characteristics

As shown in Table 1a, hepatic steatosis and anomalous portal venous or biliary anatomy were similar among the two groups. The duration of surgery (614.9 ± 117.3 vs. 771.5 ± 204.4; *p*=0.0001) and the blood loss (426.4 ± 229.8 vs. 764.2 ± 427.8; *p*=0.0001) were lower in the triple C group compared to the CS group. The “conversion to open” rate was lower among the triple C group (3.8% vs. 12.1%; *p*=0.07), although it did not reach statistical significance. In the CS group, out of a total of four conversions, two were due to bleeding during transection, one for lack of intra-abdominal space, and one for unable to progress in portal due to complex anatomy. In the triple C group, two were converted due to lack of intra-abdominal space, one for bleeding during transection, and one for unable to progress.

Postoperatively, as shown in Table 1b, the donor biliary leak rate was significantly lower among the triple C group compared to the CS group (3/104, 2.9% [all drainage] vs. 5/33, 15.2% [4 drainage, 1 ERC and stenting]; *p*=0.009). One donor in the CS group had a stricture of the bile duct which was successfully remodeled with endoscopic stenting in 7 months. The length of hospital stay was lower in the triple C group compared to the CS group (5.2 ± 0.7 vs. 6 ± 1.4 days; *p*=0.0001). There was no difference in the other morbidity after surgery among the two groups. All donors are currently well at home. During the initial 20 RDH cases in the learning curve, there were two (15%) biliary leaks and one stricture at 2 months among the donors.

### 4.3. Recipient Perioperative Characteristics

The recipients were evenly matched in both groups with respect to their demographics, Child–Turcotte–Pugh (CTP) score/model for end-stage liver disease (MELD), cold ischemia time (CIT), warm ischemia time (WIT), and GRWR. Multiple (> 1) biliary anastomoses were commoner (20.2% vs. 15.2%) among the triple C group, though not statistically significant (Table 2a).

Postoperatively, the biliary complication rate (those needing intervention beyond simple drainage) was lower among the triple C group compared to the CS group although not statistically significant (10.6% vs. 15.1%; *p*=0.537). There were no differences in other post-transplant morbidity or mortality among the two groups (Table 2b).

## 5. Discussion

We describe a novel technique during total RRDH which enables a bloodless view during division of the RHD that safeguards the RHD, while ensuring a secure donor HD stump and preventing compromise of the remnant (left) HD. A clear view enables precise division of the RHD along with its HPGS complex, resulting in a healthy, well-vascularized RHD and an adequate stump. This obviates the need to apply pressure, diathermy, or suture to the bleeding points, which can sometimes traumatize the RHD and/or the donor stump and/or compromise their blood supply.

So far, two conventional techniques have been described in the literature for HDD in minimally invasive DH/RRDH: the CS and “clip and cut” methods. In one study, the incidence of biliary complications with the CS technique was reported to be 4.5% among donors [2], whereas Rho et al. reported 0% donor biliary complications with the CS method [3].

In the early part of our series, we observed a relatively high incidence of biliary complications among donors with the CS technique, wherein the RHD was divided by scissors followed by closure of the stump by a continuous 6-0 PDS suture. We observed that the field often became quite bloody with this method, and the bleeding needed to be controlled with pressure, diathermy, or suturing. This may have led to an increased incidence of biliary complications in donors and recipients. To reduce the biliary complication rate, we devised the triple C method (described above and shown in the accompanying video clip) in which the RHD was clipped, followed by temporary clamping of the RHA to prevent bleeding from the divided hepatic duct and the hilar plate. Then, the RHD was divided. After this, a second Hem-o-lok® clip was applied on the remnant (left) side of the remaining hilar plate, and a temporary laparoscopic clip was applied on its right side, following which the hilar plate was divided with scissors between the two clips. After having secured 3 areas that could potentially bleed—the remnant HD stump, and both sides of the hilar plate, the RHA clamp was released after 5-6 min. The latter has previously been shown to be harmless in open donor hepatectomy in the setting of intentional ischemic preconditioning [7]. With the triple C modification, we were able to minimize the donor biliary complication rate, and the 3 donors that had a bile leak just needed simple drainage for up to a week.

Among recipients of RRDH, the biliary leak has been reported in 16.9% and strictures in 12.4% with the CS technique [2]. In another study, the reported biliary stricture rate was 15.8% among recipients with the “clip and cut” method [3]. In our series, there was a trend toward lower recipient biliary complication rate (down from 15% to 10%) by switching from the CS to the “clip, clamp, and cut” technique (triple C). This was despite a higher proportion of multiple biliary anastomoses among the triple C group. We and others have previously reported similar recipient biliary complication rates for open donor hepatectomy [8, 9].

Lower operating times, less blood loss, and early discharge among the donors of the triple C group were likely to be attributable to improved surgical expertise with experience.

## 6. Limitation

One of the limitations of the study was its retrospective nature. However, as the new technique served us well for the first few cases, we felt it was an improvement and could not justify having a control group for a future prospective study.

Propensity score matched comparison was not performed as it would make the CS cohort too small to compare and conclude.

Another limitation was that the triple C technique was later in the learning curve which could have been a confounding factor in the reduction of postoperative biliary complications in the triple C group.

In conclusion, we describe a novel technique of HDD during total RRDH which provides a clear view around the HDC and enables clean, precise, and bloodless division of the graft hepatic duct, likely resulting in lower donor, and potentially lower recipient biliary complication rates. The simplicity and reproducibility of the technique make it ideal for widespread adoption.

## Figures and Tables

**Table 1a:** Donor pre- and intraoperative characteristics in the CS and the triple C groups.

		**CS group (33)**	**Triple C group (104)**	***p* value**
Age (years)		28.5 ± 8.4	31.2 ± 8.4	0.10

Gender, F/M		17/16	54/50	0.96

BMI (kg/m^2^)		23.9 ± 2.9	24.1 ± 3.1	0.75

Steatosis > 10%		1 (3%)	4 (3.8%)	0.82

Conversion to open		4 (12.1%)	4 (3.8%)	0.07

Operating time (minutes)		771.5 ± 204.4	614.9 ± 117.3	0.0001⁣^∗^

Portal vein type	A	32 (97%)	101 (97.1%)	0.71
	B	0 (0%)	1 (1%)	
	C	1 (3%)	1 (1%)	

Biliary anatomy	A1	22 (66.7%)	66 (63.5%)	0.81
	A2	5 (15.2%)	14 (13.5%)	
	A3	4 (12.1%)	13 (12.5%)	
	A4	1 (3%)	9 (8.7%)	
	A5	1 (3%)	1 (1%)	
	B1	0 (0%)	1 (1%)	

Estimated blood loss (mL)		764.2 ± 427.8	426.4 ± 229.8	0.0001⁣^∗^

⁣^*∗*^Indicates statistically significant values (*p* < 0.05).

**Table 1b:** Donor postoperative outcome in the CS and the triple C groups.

		**CS group (*n* = 33)**	**Triple C group (*n* = 104)**	***P* value**
Clavien–Dindo grade	I/II/IIIA	11 (33%)	21 (20%)	0.34
	IIIB, IV	1⁣^∗^ (3%)	3⁣^∗∗^ (2.9%)	0.89

Re-exploration		1 (3%)	1 (1%)	0.57

Postop bile leak—biloma		5 (15.2%), (drainage 4, ERC-stent 1)	3 (2.9%), (drainage 3)	0.009⁣^∗^

Postop biliary stricture		1 (3%)	0 (0%)	0.075

Hospital stay in days		6 ± 1.4	5.2 ± 0.7	0.0001⁣^∗^

*Note:* Two donors were shifted to ICU: one for hypoxia due to pneumonitis and one had supraventricular tachycardia on ECG, which were evaluated and managed conservatively.

⁣^∗^Re-explored for bile leak on day 3.

⁣^∗∗^Clavien–Dindo grade ≥ IIIB: re-exploration for rectus sheath hematoma (*n* = 1).

**Table 2a:** Recipient pre- and intraoperative characteristics in the CS and the triple C groups.

		**CS group (*n* = 33)**	**Triple C group (*n* = 104)**	***P* value**
Age (years)		52.6 ± 10.4	51.3 ± 11.2	0.56

Gender, F/M		9/24	20/84	0.32

BMI (kg/m^2^)		25.4 ± 3.3	25.1 ± 4.2	0.67

CTP		8.6 ± 2.2	8.8 ± 2.2	0.75

MELD		19.6 ± 16	18 ± 11	0.52

CIT (minutes)		104.4 ± 29.9	104.1 ± 35.1	0.97

WIT (minutes)		34.7 ± 11.3	31.1 ± 14.2	0.19

GRWR		1 ± 0.2	1 ± 0.2	0.52

No. of biliary anastomoses	1	28 (84.8%)	83 (79.8%)	0.57
	2	5 (15.2%)	18 (17.3%)	
	3	0 (0.0%)	3 (2.9%)	

Abbreviations: BMI, body mass index; CIT, cold ischemia time; CTP, Child–Turcotte–Pugh score; GRWR, graft-to-recipient weight ratio; MELD, model for end-stage liver disease; WIT, warm ischemia time.

**Table 2b:** Recipient postoperative outcome in the CS and the triple C groups.

	**CS group (*n* = 33)**	**Triple C group (*n* = 104)**	***p* value**
Bile leak—biloma needing drainage only	1 (3.05%)	2 (2%)	0.64

Biliary stricture—early (< 30 d)	1⁣^∗^ (3%)	2 (1.9%)	0.537
Biliary stricture—late	4⁣^∗∗^ (12.1%)	9⁣^∗∗^ (8.7%)	

Hepatic artery thrombosis (HAT)	1 (3%)	1 (1%)	0.38

Hospital stay in days	14.1 ± 5.5	14.5 ± 11	0.84

Hospital mortality (index admission/30 days, whichever was longer)	2 (6.1%)	7 (6.7%)	0.89

⁣^∗^This patient had early biloma which led to stricture.

⁣^∗∗^One patient in the CC and two in the triple C groups who had early bilomas.

## Data Availability

The data that support the findings of this study are available from the corresponding author (K.S.Y.) upon reasonable request.

## Supporting Information

Video showcasing the CS technique (older) and our novel triple C technique for HDD in RDH.
