# Supplementary material for: Hepatic Duct Division During Robotic Living Donor Hepatectomy: A Comparison Between the Novel Triple C (Clip–Clamp–Cut) and the Cut–Suture Techniques
Source: J Transplant. 2024 Oct 15;2024:8955970. doi: 10.1155/2024/8955970 (PMC11502124; doi:10.1155/2024/8955970)
Supplement: Supporting Information — Additional supporting information can be found online in the Supporting Information section. [file 8955970.f1.docx]

**Hepatic duct division during robotic living donor hepatectomy: a comparison between the novel triple C (clip-clamp-cut) and the cut-suture techniques.**

Arvinder S. Soin^1^, Kamal S. Yadav^1^, Fysal Valappil^1^, Nikhitha Shetty^1^, Raghav Bansal^1^, Suchet Chaudhary^1^, Ankur Gupta^1^, Amit Rastogi^1^, Prashant Bhangui^1^

1- Institute of Liver Transplantation and Regenerative Medicine, Medanta The Medicity Hospital, Gurugram, India.

**Corresponding Author:**

Kamal S. Yadav,

Consultant and Robotic-incharge,

Liver transplant surgeon,

Institute of Liver Transplantation and Regenerative Medicine, Medanta The Medicity Hospital, Gurugram, India.

Email: [dockamalyadav@gmail.com](mailto:dockamalyadav@gmail.com)

Mob. No: +91 9920375758.

**Video link:**

<https://drive.google.com/file/d/1CsLcJkzpV5eSLKV9o-Cvo56Go1SX8T22/view?usp=share_link>
